# Dosimetric effect of respiratory motion on volumetric‐modulated arc therapy‐based lung SBRT treatment delivered by TrueBeam machine with flattening filter‐free beam

**DOI:** 10.1120/jacmp.v14i6.4370

**Published:** 2013-11-04

**Authors:** Xiang Li, Yong Yang, Tianfang Li, Kevin Fallon, Dwight. E Heron and, M. Saiful Huq

**Affiliations:** ^1^ Department of Radiation Oncology University of Pittsburgh Medical Center Pittsburgh PA USA

**Keywords:** stereotactic body radiotherapy, volumetric‐modulated arc therapy, 4D CT, deformable registration, dynamic MLC, interplay effect

## Abstract

The purpose of this study is to investigate the interplay effect between dynamic MLC movement and tumor respiratory motion in volumetric‐modulated arc therapy (VMAT)‐based lung SBRT treatment delivered by the flattening filter‐free (FFF) beam of a Varian TrueBeam machine. Six lung cancer patients with tumor motions ranging between 0.5–1.6 cm were recruited in this study. All patients underwent 4D‐CT scan with audiocoaching. A two‐arc VMAT plan was retrospectively generated using Varian's Eclipse planning system for each patient. To explicitly describe the interplay effect, the contributions of each control point in the original static VMAT plans to each respiratory phase were calculated, and then ten new VMAT plans corresponding to different respiratory phases were generated and imported back into Eclipse planning system to calculate the radiation dose based on the CT images of related respiratory phase. An in‐house 4D dose calculation program with deformable registration capacity was used to calculate the accumulative 4D dose distribution of the targets. For all patients, the PTV coverage dropped significantly with increased respiratory motion amplitude. However, V100 and D90 of the GTV and GTV+5mm, which mimic the target with setup error of less than 5 mm, were either unchanged or slightly increased up to 1.2%, and the variations of their minimum doses were less than 3.2%. Our results indicated that for VMAT‐based lung SBRT treatment delivered by FFF beam of TrueBeam machine, the impact of interplay effects on target coverage is insignificant, as long as a sufficient margin was given.

PACS number: 87.53.Ly

## I. INTRODUCTION

Compared to conventional radiotherapy, hypofractionated stereotactic body radiotherapy (SBRT) has been demonstrated to be a more effective treatment modality for stage I non‐small cell lung cancer patients with improved local tumor control and survival rate.^1^, ^2^ SBRT technique requires a higher biological effective dose (BED) delivered to lung tumors while sparing radiation dose to the surrounding critical structures to minimize the complication occurrence.^(3)^ To achieve this dosimetric requirement, intensity‐modulated radiotherapy (IMRT) has been widely adopted in clinic practice.^(4)^ In recent years, volumetric‐modulated arc therapy,^(5)^ which can produce a treatment plan with comparable quality to a conventional IMRT plan, has been developed and rapidly implemented in lung SBRT treatment due to significantly reduced MU, highly efficient delivery, and potentially better dose conformity.^(6)^

However, in either IMRT or VMAT treatments, there is always a concern about the interplay effects between the dynamic multileaf collimator (MLC) movement and tumor respiratory motion which might create undesired hot or cold dose spots inside the lung tumor. Jiang et al.^(7)^ investigated intrafractional organ motion effects in conventional lung IMRT treatments and concluded that the interplay effect could result in up to 18% dose variation inside lung tumor for single fraction treatment, but became less than 1%~2% after 30 fractions. Seco et al.^(8)^ investigated effects of organ motion on conventional IMRT treatments with segments of few monitor units and observed that for small MU segments, nonnegligible biological effects can be incurred. Those conclusion might not be applicable in lung SBRT treatment, since a much higher radiation dose was delivered in a single fraction with total of only 3~5 fractions. Kang et al.^(9)^ investigated respiratory motion induced dosimetric effects on IMRT based lung SBRT treatment. Their simulation study indicated that, for typical tumor geometries and respiratory amplitudes, changes in target coverage are minimal but can be significant for larger amplitudes, faster beam delivery, more highly modulated fields, and smaller field margins. Ong et al.^(10)^ investigated the dosimetric impact of interplay effect on RapidArc lung SBRT treatment using Varian Trilogy machine with phantom measurements and film dosimetry. They found that the interplay between DMLC and tumor motion is not significant for single‐fraction treatments when RapidArc is delivered with two different arcs. Rao et al.^(11)^ conducted a 4D computerized tomography (CT)‐based study to investigate the influence of tumor motion on dose delivery in lung SBRT treatment using both IMRT and VMAT plan based on Elekta Synergy machine. This study enabled dose‐volume histogram (DVH)‐based analysis on target coverage, and concluded that both VMAT and IMRT plans experienced negligible interplay effects between MLC sequence and tumor motion in lung SBRT treatment. It should be mentioned that zero setup errors were assumed in above studies.

Recently Varian released their new generation linear accelerator, TrueBeam machine, which is equipped with flattening filter‐free (FFF) beams to deliver ultrahigh dose rate for SBRT treatment. The purpose of this study is to investigate the interplay effect between dynamic MLC movement and tumor respiratory motion for VMAT‐based lung SBRT treatment delivered by FFF beams of TrueBeam machine. In addition, the effect of setup error on target coverage was also included in this study.

## II. MATERIALS AND METHODS

### A. Patient selection

Six non‐small cell lung cancer (NSCLC) patients were retrospectively selected for this study. All patients underwent 4D CT scan using GE LightSpeed 16 scanner (GE Healthcare, Waukesha, WI) under audiocoaching, and the respiratory cycle was set as 3 to 5 seconds based on their natural breathing pattern. For each patient, ten sets of phase‐sorted CT images with a slice thickness of 2.5 mm were acquired using GE advantage 4D workstation (GE Healthcare) and imported into Varian Eclipse treatment planning system (Varian Medical Systems, Palo Alto, CA) for further processing. Gross tumor volumes (GTV) were drawn on CT images at all respiratory phases, and then were combined together to generate the internal tumor volume (ITV) on the CT images at phase 50% which represents the end of exhale stage. Afterwards, a 5 mm uniform margin was added to generate the planning tumor volume (PTV). The size of PTV ranged from 34.2 cc to 157.3 cc, with a mean volume of 82.9 cc, and the respiratory motion amplitudes ranged from 0.5 cm to 1.6 cm.

### B. VMAT plan for lung SBRT

**Figure 1:**
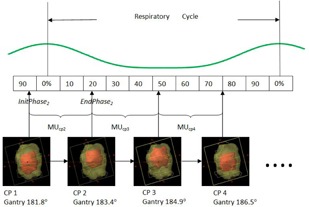
Illustration of interplay effect and contribution of each control point to different respiratory phase (cp stands for control point; yellow and red structure represent PTV and GTV).

Varian Eclipse treatment planning system was used to generate all VMAT plans based on 6 MV FFF beam of TrueBeam machine equipped with high‐definition 120 multileaf collimators with central leaf thickness of 2.5 mm. For each VMAT plan, two coplanar arcs with at least 200° rotation were adopted, and the collimator angles of 20° and 340° were used for each arc. The nominal dose rate of this beam is 1400MU/min. Moreover, in each VMAT plan, at least 95% of PTV was covered by the prescribed dose (60 Gy in 3 fractions), and the dose limits for all the surrounding critical structures were all within the tolerance. Radiation dose was calculated by anisotropic analytical algorithm (AAA) with inhomogeneity correction, and plan evaluations were performed based on the CT images at phase 50%. Those VMAT plans and the corresponding doses were referred to as static plans and static 3D doses. As illustrated in Fig. 1, the dynamic MLC shape changes with different gantry angles, while the tumor moves at the same time. Due to this interplay effect, the static dose distribution generated above did not reflect the actual dose distribution of the targets. In order to calculate the true doses, referred to as 4D dose in this study, we adopted a similar approach used by Rao et al.^(11)^ First, an original VMAT plan was exported out from Eclipse TPS via DICOM RT export function, and then was processed by in‐house software to generate 4D VMAT plan with DICOM format for each respiratory phase. The goal of each 4D VMAT plan is to force the original static VMAT plan delivering radiation during the treatment only at the time which belongs to the corresponding respiratory phase. In Varian Eclipse treatment planning system, each arc beam of a VMAT plan consists of a series of control points (cp). Each control point was determined by a specific gantry angle, a static beam aperture shaped with MLC, and a cumulative dose coefficient (CDC), which corresponds to the amount of MU delivered at this angle, as defined in Eq. (1).

(1) $$MU_{cpi}=TotalMU*(CDC_{cpi}-CDC_{cpi-1})$$

Due to the respiratory motion during the SBRT treatment, as seen in Fig. 1, each control point could contribute to one or more respiratory phases. Assume that the initial respiratory phase of control point i is ${\text{InitPhase}}_{i}$, then the ending respiratory phase of control point i could be calculated as follows:

(2) $$EndPhase_{i}=InitPhase_{i}+\frac{MU_{cpi}*Doserate_{cpi}}{T}$$

where *T* is the time duration of each respiratory phase, which is a tenth of the respiratory cycle. Thus, the amount of MU contributing to each respiratory phase (${\text{MUperphase}}_{\text{cpi}}$) at control point i between ${\text{InitPhase}}_{i}$ and ${\text{EndPhase}}_{i}$ was given as:

(3) $$MUperphase_{cpi}=\frac{MU_{cpi}}{Endphase_{i}-InitPhase_{i}}$$

**Figure 2:**
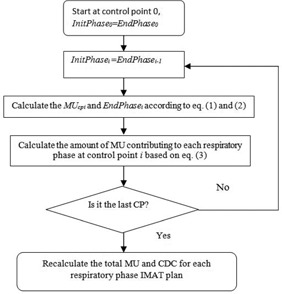
Flowchart showing how to generate 4D VMAT plans.

The MU at control point i for other respiratory phases which are not belonging to the phase interval between ${\text{InitPhase}}_{i}$ and ${\text{EndPhase}}_{i}$ will be set to zero. According to Eq. (2), ${\text{InitPhase}}_{i}$ and ${\text{EndPhase}}_{i}$ may not be an integer. If this is the case, a portion of ${\text{MUperphase}}_{\text{cpi}}$ was assigned to the initial and ending respiratory phases. The initial phase of control point 0 was randomly selected between phase 0% and phase 90%. Figure 2 shows the flowchart of calculating the amount of MU contributing to each respiratory phase for all control points. Once the MUs contributing to all respiratory phases at each control points were calculated, the total MU and the corresponding CDC of each control point for different respiratory phases could be calculated accordingly. Thus, ten new VMAT plans related to different respiratory phases could be generated. It should be noted that the beam aperture at each control point still remained the same for all new VMAT plans; therefore, all ten VMAT plans have same DMLC sequence as the original 3D VMAT plan, and the only difference between the different VMAT plans is the amount of MU delivered at each control point. Afterward, ten new VMAT plans with DICOM format were imported back into an Eclipse treatment planning system via DICOM RT import function, and then were linked to the CT images at the corresponding respiratory phases to calculate the dose distribution.

### C. 4D dose calculation

**Figure 3:**
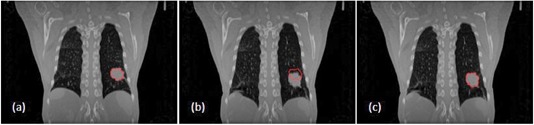
Automatic target contour based on image deformable registration.

The general principle of 4D dose calculation is to perform a conventional 3D dose calculation based on each phase‐sorted 4D CT dataset with the corresponding treatment plan that incorporates the interplay effects, then utilize a deformable image registration to deform the dose matrix from a reference phase to other respiratory phases, and accumulate the doses altogether to obtain a 4D dose distribution. The key of 4D dose calculation is to trace the deformation trajectory of each voxel during respiratory cycle. An in‐house intensity‐based automatic deformable registration software,^(12)^ based on a “demons” algorithm,^(13)^ was adopted in this study. Assume that m and f denote the moving image and the fixed image, respectively. Thus, the deformation field *D* is given by following equation:

(4) $$\vec{D}=-(m-f)\frac{\nabla m+\nabla f}{{\left\|\nabla m\right\|}^{2}+{\left\|\nabla f\right\|}^{2}+\kappa {\left(m-f\right)}^{2}}$$

where κ is the normalization factor which adjusts the force strength. To accelerate the convergence speed, a multiresolution strategy was adopted. After each iteration, a Gaussian low‐pass filter was applied to maintain a smooth deformation field. Figure 3 shows an example to demonstrate the performance of the deformable registration algorithm described above. Figures 3(a) and 3(b) are CT images at end‐exhale and end‐inhale stage, respectively. Due to the respiratory motion, the tumor contour drawn at end‐hale stage shown in Fig. 3(a) does not match the tumor position at end‐inhale stage, as seen in Fig. 3(b). Figure 3(c) shows the end‐inhale CT image with the automatic tumor contour deformed by the above deformable registration method from the tumor contour at end‐exhale stage.

Deformable registration builds the voxel to voxel correspondence between the moving image and fixed image. Given such information, the accumulative dose that the moving target receives during respiration can be calculated by using the following method. First, the radiation dose distribution is calculated based on the CT images at each respiratory phase with the corresponding VMAT plan, as described above. Thereafter, the accumulative radiation dose $D_{i}$ of voxel i in the CT images of phase 50% can be computed as

(5) $$D_{i}=\sum_{j=1}^{N}D_{i^{\prime }j}$$

where *N* is the number of breath phases, and voxel *i'* is the trajectory of the voxel *i* from phase 50% to the breath phase $j.D_{i,j}$ is the dose of voxel *i'* received at breath phase j.

It is worthy to emphasize that dose deformation is a big challenge of 4D radiotherapy in general and highly depends on the accuracy of image deformable registration, which is mathematically an ill‐posed problem. Vinogradsky et al.^(14)^ have validated the accuracy of 4D dose calculation based on a deformable phantom and found out that, if patient's breathing is reproducible, the 4D dose calculation could be accurate to within clinically acceptable standards. Our experience is that the deformable registration method used in this study can archive a high accuracy for a situation where there is a high image intensity contrast between target and its surrounding structures, such as the case shown in Fig. 3. When the lung tumor was attached to chest wall or adjacent to the mediastinum, the accuracy of deformable registration will be degraded.

## D. Plan evaluation

It should be pointed out that zero setup errors and a stable respiratory motion pattern were assumed in this study. Since the PTV volume was defined as ITV plus 5 mm margin, which was used to compensate the setup errors and irregular breath pattern changes such as respiratory motion baseline drift, the GTV structure alone was not enough to evaluate the target dose coverage when there are setup errors present. Assuming that a random setup error along any directions in 3D space was less than 5 mm, in order to incorporate the setup error effect into this study, a new virtue structure “GTV+5mm”, which is defined as GTV plus 5 mm uniform margin, was also introduced. To quantitatively evaluate the interplay effects between dynamic MLC and lung tumor motion on target coverage, dose‐volume histogram (DVH) analysis along with the following quantitative dosimetric parameters were adopted: (1) V100, the percentage of a target volume receiving the prescribed dose; (2) D90, the percentage dose covering 90% of the target; (3) MD, the minimum dose received by the target. For lung dose evaluation, the following parameters were used: (1) V5, the percentage of lung volume receiving 5 Gy; (2) V10, the percentage of lung volume receiving 10 Gy; (3) V20, the percentage of lung volume receiving 20 Gy; (4) Mean Dose, the mean dose of lung volume received.

## III. RESULTS

**Figure 4:**
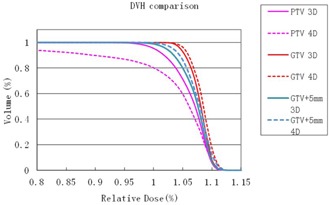
DVH comparisons between static 3D dose and the 4D dose distribution for a real patient with 1.6 cm respiratory motion.

Figure 4 shows DVH comparisons between 3D and 4D dose distribution for a real patient with a respiratory motion of 1.6 cm. The pink lines, red lines, and blue lines represent the DVH curves of PTV, GTV, and GTV+5mm, respectively. The straight lines and dashed lines represent the 3D dose distribution and the calculated 4D dose distribution, respectively. Figure 3 has clearly shown that the coverage of PTV volume dropped significantly due to the interplay effect between dynamic MLC movement and tumor motion. The V100, D90, and MD of the PTV volume dropped 15.4%, 13.1%, and 43.3%, respectively. However, the coverage of GTV was not affected by the interplay effects. V100 and MD of the GTV volume remained nearly unchanged, and D90 slightly increased about 0.6%. A better coverage of GTV+5mm was observed in this case. V100, D90, and MD of GTV+5mm increased 0.6%, 0.9%, and 2.9%, respectively.

**Figure 5:**
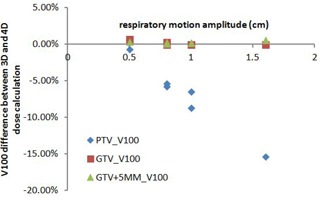
Scatter chart of V100 differences between static 3D dose and the 4D dose calculation of PTV, GTV, and GTV+5mm for all six patients.

Figure 5 shows a scatter chart of V100 differences between 3D and 4D dose calculation of PTV, GTV, and GTV+5mm structures for all six patients. The dots of blue diamond, red square, and green triangle represent the PTV, GTV, and GTV+5mm structures, respectively. X‐axis is the respiratory motion amplitude, and y‐axis is the V100 coverage variation between 3D and 4D dose calculation. Due to the interplay effect, the V100 coverage of PTV dropped from 0.7% to 15.4% with the increase of the respiratory motion amplitude from 0.5 cm to 1.6 cm. However, V100 coverage differences between 3D and 4D dose calculation for GTV and GTV+5mm were less than 0.7%, regardless of the amplitude of the respiratory motion for all six patients.

**Figure 6:**
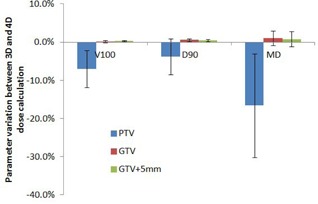
Statistical analyses of parameter variations between static 3D dose and 4D dose calculation of PTV, GTV, and GTV+5mm structures.

Figure 6 shows the statistical analyses of parameter variations between 3D and 4D dose calculations of PTV, GTV, and GTV+5mm structures for all six patients due to the interplay effect between dynamic MLC movement and tumor motion. The mean variation and its standard deviation of each parameter for all six patients is indicated by a wide bar and an error bar, respectively. Compared to the 3D static dose, the interplay effect reduced the mean of V100, D90, and MD for PTV volumes by −7.1%,−3.8%, and −16.6%, respectively. The standard deviations of those parameters were 4.8%, 4.7%, and 13.6%, respectively. In comparison, the interplay effect did not significantly compromise the coverage of GTV and GTV+5mm volumes. The mean variations and its standard deviation of V100, D90, and MD for GTV and GTV+5mm were less than 1%, except that the standard deviations of MD variation of GTV and GTV+5mm were about 2%.

**Figure 7:**
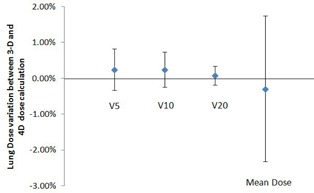
Lung dose variation between 3D dose and the 4D dose distribution for all six patients.

Lung dose distribution is always a great concern for any type of lung SBRT treatment. Figure 7 shows the lung dose variations between 3D and 4D dose calculations due to the interplay effects. The blue dots shown in Fig. 7 from left to right correspond to the mean variations of V5, V10, V20 and mean lung dose between 3D and 4D dose calculations for all six patients, respectively, and the error bars indicate the standard deviation of each parameter. It can be clearly seen that the mean variations of V5, V10, V20, and lung mean dose were all less than 0. 4%, and their standard deviations were less than 0.6% except for the lung mean dose, for which the standard deviation was about 2.0%. The results suggested that the interplay effects on lung dose distribution were insignificant.

## IV. DISCUSSION

Our results suggest that the interplay effects between dynamic MLC and moving target in lung SBRT treatment delivered by TrueBeam FFF beams did not compromise the GTV coverage. This finding is consistent with other studies' results.^9^, ^10^, ^11^ In the study by Rao et al.,^(11)^ VMAT plans were generated with less intensity modulation using ${\text{Pinnacle}}^{3}$ SmartArc inverse planning module (Philips Healthcare, Andover, MA). They indicated that the small interplay effect might be due to the fact that most of the segments in a VMAT or IMRT plan contain an aperture shape conformed to the PTV, and there are only a few segments with MLC leaves blocking part of the target in order to spare critical structures. In comparison, as seen in Fig. 1, there are target blockages by MLC on every segment, which means that the degree of intensity modulation was much stronger in our study. Our results suggested that the interplay effects might be irrelevant to the types of MLC and the degree of intensity modulation for VMAT‐based lung SBRT treatment. However, it is worthy to emphasize again that a zero setup error and stable respiratory motion pattern were assumed in the above studies. It might raise a concern that setup error could compromise this unaffected target coverage. By taking a setup error into account and assuming that this error is within a 5 mm range, the coverage of GTV+5mm structure represented more realistic target coverage with the presence of setup error which is less than 5 mm in all directions. Our results demonstrated that the target coverage would not be compromised by the simultaneous interplay effect and setup error as long as a setup error is within the margin.

Our results also demonstrated that the PTV coverage deteriorated rapidly with the increase of the magnitude of the respiratory motion. It was obvious that the larger the respiratory motion, the greater the chance of the PTV volume moving out of treatment fields. This result insinuated that if there are not enough margins around the GTV volume due to a setup error and an irregular respiratory motion, it is very likely that the target will be underdosed by the interplay effect. In a routine lung SBRT treatment, taking an on‐board cone‐beam CT image is a standard procedure to increase positional accuracy of the patient. The accuracy of such imaging system for a static target is about 1 mm. However, due to the respiratory motion and a slow scanning nature of CBCT technique, there are significant motion artifacts presented in lung CBCT image, as demonstrated by Vergalasova et al.^(15)^ Those artifacts are highly depended on the pattern of the respiration motion. When the time duration of the exhale stage is much larger than that of the inhale stage, the moving target in CBCT image is more closely represented by phase 50%. However, if the time spans of the exhale and inhale stages of a respiratory motion are similar, the target characteristics determined by CBCT image are rather complicated. It was neither the target at phase 50% nor the union of the target from all respiratory phases (ITV), and the image contrast between the target and its surrounding tissues was also significantly reduced. The accuracy of localizing the target position for such moving target based on conventional slow‐scanning CBCT images should be considered in the PTV volume. In our clinic, on‐board kV fluoroscopy imaging has been used to facilitate the target positioning and the gating phase verification. For a visible target, this technique is capable of detecting whether, during the beam‐on period, the target always locates within the field aperture, which represents the PTV volume, or not. A more accurate way to position the moving target for lung SBRT treatment is to use 4D CBCT imaging which is capable of acquiring phase‐resolved CBCT images, which currently is still not available in clinical practice. Many researchers have put a lot of effort into developing this technique, and have achieved significant progress to make it available for clinical use in the near future.^16^, ^17^, ^18^, ^19^, ^20^

Due to the nature of the 4D CT scan, there were only ten respiratory phases, and a stable respiratory motion pattern was assumed in this study. The main limitation of such assumptions is that irregular respiratory motion patterns were not taken into account. Several studies^21^, ^22^ have investigated the dosimetric impacts of irregular respiratory motion on lung tumor coverage and have shown that the irregular respiratory motion might deteriorate the target coverage. To mitigate the occurrence of respiratory irregularity, several techniques have been developed. Audiovideo feedback technique^(23)^ has been shown to be an effective way of achieving a stable respiratory pattern. Gating^(24)^ is another popular technique to prevent the radiation delivery when an irregular breathing pattern occurs, such as coughing or sneezing, but at the expense of longer treatment time. Real‐time tumor tracking, along with appropriate MLC^25^, ^26^ or couch^(27)^ compensation technique, is a more promising approach to treat a moving target, and has been a very active research topic in recent years. The key to the success of this technique is to seek an effective way to track the tumor in real time using either external respiratory surrogates^(28)^ or on board kV and MV imaging.^29^, ^30^

## V. CONCLUSIONS

In summary, for lung SBRT VMAT treatments delivered by the FFF beam of TrueBeam linear accelerator, the impact of interplay effect between the dynamic MLC movement and tumor respiratory motion on target coverage is insignificant, as long as a sufficient margin was given. On‐board 4D CBCT imaging and effective real‐time tumor tracking techniques are essential to further improving the accuracy of the target localization and ensuring a more accurate treatment delivery.
